# A Model-Based Framework to Identify Optimal Administration Protocols for Immunotherapies in Castration-Resistance Prostate Cancer

**DOI:** 10.3390/cancers14010135

**Published:** 2021-12-28

**Authors:** Roberta Coletti, Andrea Pugliese, Andrea Lunardi, Orazio Caffo, Luca Marchetti

**Affiliations:** 1 Fondazione The Microsoft Research, University of Trento Centre for Computational and Systems Biology (COSBI), 38068 Rovereto, Italy; 2 Department of Mathematics, University of Trento, 38123 Trento, Italy; andrea.pugliese@unitn.it; 3 Department of Cellular, Computational and Integrative Biology (CIBIO), University of Trento, 38123 Trento, Italy; andrea.lunardi@unitn.it; 4 Medical Oncology Department, Santa Chiara Hospital, 38122 Trento, Italy; orazio.caffo@apss.tn.it

**Keywords:** prostate cancer, immunotherapy, optimal administration protocols, ipilimumab, sipuleucel-T, mathematical modeling, ordinary differential equations (ODEs), computational biology, optimization problems, toxicity function

## Abstract

**Simple Summary:**

Although *ipilimumab* has been approved for the treatment of many types of cancer, most prostate cancer patients seem to not respond well to the therapy. Here, we mathematically investigate the clinical relevance of *ipilimumab*, as mono-therapy and in combination with the dendritic cell vaccine *sipuleucel-T*, for the treatment of castration-resistant prostate cancer patients. The employed optimization problem, which incorporates a function of toxicity depending on the drug concentration, establishes precise protocols of administration of *ipilimumab* to control or eradicate prostate tumor, and defines how changing of key parameters affects the outcome. Overall, this mathematical study can help in optimizing the clinical use of *ipilimumab* for the effective treatment of castration-resistant prostate cancer patients.

**Abstract:**

Prostate cancer (PCa) is one of the most frequent cancer in male population. Androgen deprivation therapy is the first-line strategy for the metastatic stage of the disease, but, inevitably, PCa develops resistance to castration (CRPC), becoming incurable. In recent years, clinical trials are testing the efficacy of anti-CTLA4 on CRPC. However, this tumor seems to be resistant to immunotherapies that are very effective in other types of cancers, and, so far, only the dendritic cell vaccine *sipuleucel-T* has been approved. In this work, we employ a mathematical model of CRPC to determine the optimal administration protocol of *ipilimumab*, a particular anti-CTLA4, as single treatment or in combination with the *sipuleucel-T*, by considering both the effect on tumor population and the drug toxicity. To this end, we first introduce a dose-depending function of toxicity, estimated from experimental data, then we define two different optimization problems. We show the results obtained by imposing different constraints, and how these change by varying drug efficacy. Our results suggest administration of high-doses for a brief period, which is predicted to be more efficient than solutions with prolonged low-doses. The model also highlights a synergy between *ipilimumab* and *sipuleucel-T*, which leads to a better tumor control with lower doses of *ipilimumab*. Finally, tumor eradication is also conceivable, but it depends on patient-specific parameters.

## 1. Introduction

Prostate cancer (PCa) is one of the most common tumor in male population [1]. Although organ-confined tumors are successfully controlled by surgery or radiotherapy [2], a quote of patients experiences biochemical progression and most of them ultimately develop distant metastases. Androgen deprivation therapy represents the treatment cornerstone of advanced disease and provides a variable period of disease control [3]. Nevertheless, all the patients show progressive disease and achieve a condition of castration-resistance. In this disease state several agents demonstrated to be able to significantly prolong the patients’ survival: chemotherapeutics (docetaxel, cabazitaxel), androgen-receptos signaling inhibitors (ARSI), radiopharmaceuticals (radium 223) [4]. In addition, new generation ARSI are tested for advanced prostate cancer, such as apalutamide, darolutamide, and enzalutamide in non metastatic castration resistant prostate cancer (CRPC) [5], while abiraterone, apalutamide, and enzalutamide have been employed in metastatic castration sensitive prostate cancer (CSPC) treatments [6]. However, there are no efficacious therapeutic options for the treatment of metastatic CRPC. Immunotherapy represents an innovative anticancer strategy which recently led to unprecedented improvements in the prognosis of several tumors, such as lung cancer, renal cancer, melanoma, head and neck cancer [7]. The most widely used immunotherapies are based on the administration of checkpoint inhibitors which exert their anticancer activity by targeting immune checkpoints as PD1 or PDL1. In addition, anti-CTLA4 drugs, such as *ipilimumab*, demonstrated their efficacy in some tumors, such as melanoma [8].

PCa is usually considered as a “cold” tumor, with an immuno-suppressive microenvironment. Nevertheless, different immunotherapy-based strategies have been tested in prostate cancer patients, including vaccine-based therapies and CTLA-4 and PD1-PDL1 inhibition [9]. To date, *sipuleucel-T* is the only immunotherapy agent approved for the tratment of PCa by Food and Drug Administration (FDA), but not by European Medicines Agency (EMA). On the other hand, conflicting results were obtained with *ipilimumab*. However, this drug remains a potentially active agent in this disease, and therefore clinical trials are investigating its efficacy in the treatment of PCa [10].

Mathematical models describing tumor-immune interactions can support clinical decisions. In particular, they can be employed to predict the efficacy of an immunotherapy and, therefore, can help in identifying mechanisms that need to be further investigated. There are many mathematical models of PCa. A comprehensive review [11], published in 2020, collects the main models describing PCa evolution and its interaction with immune system. Most of the models in literature include androgen-deprivation therapy, with the aim of investigating the effect of this treatment on PCa, or to find the optimal-drug delivery [12]. There are also works focusing on PCa immunotherapy, but they include only the dendritic cell vaccine *sipuleucel-T*. The first model considering more than one immunotherapy has been proposed by Peng et al. [13]. The authors developed a pre-clinical model of PCa including three different immunotherapies: the dendritic cell vaccine, an anti-Treg drug, and anti-IL-2. In a previous work, we extended this model [14], by including a more detailed description of the tumor micro-environment and two other immunotherapies, the infusion of Natural Killer cells (NK) and an Immune-Checkpoint Inhibitor (ICB). These extensions allowed us to test a large variety of possible combination therapies, by considering both their efficacy in reducing the tumor and their synergy. We also developed a model of human castration-resistant PCa including dendritic cell vaccine *sipuleucel-T* and the anti-CTLA4 *ipilimumab* [15]. This work aimed at investigating the effect of the immunotherapies on the model steady-states, in order to evaluate if they could lead to tumor eradication. Our results showed that therapies involving the drug *ipilimumab* are potentially able to make the no-tumor steady-state attractive, for some reasonable parameter values. Given these promising results, we decide to employ this mathematical model in the present work. The goal of this project is to identify the optimal administration protocol of the drug *ipilimumab*, which, administered as mono-therapy or in combination with the vaccine *sipuleucel-T*, is able to control or eradicate the tumor. To this end, we need to integrate our previous model by including information about drug toxicity, which is crucial to define a reliable administration protocol. Indeed, when patients are subject to drug administration, adverse events (AEs) can occur.

Side effects due to drug administration are often classified as moderate, i.e., grade 1 and 2, and severe, with grade ≥3. The latter are generally more intense, and they can have a longer resolution time. The AEs observed in a clinical trial are always collected and analyzed in order to estimate the drug toxicity. Sometimes, promising clinical trials have been interrupted due to unexpected side effects, and therefore, this information cannot be ignored. In the literature, there are several examples of PK/PD mathematical models defining the drug toxicity also from a molecular point of view. For example, the work by El-Masri et al. [16] describes the toxicity by considering the effect of a mixture of drugs on the human body. Similar methods have been employed also in the context of prostate cancer [17]. Even if this approach have been previously used to define optimal control problems [18], for our scope, we do not need such a detailed description, which would increase the model complexity both from mathematical and computational point of view. Another possible approach for taking into account drug toxicity in optimal control problems is based on connecting the toxicity with the drug concentration, by defining a maximum acceptable drug exposure and a critical concentration threshold, over which the drug becomes not tolerated [19,20]. However, the *ipilimumab* has not yet been approved for PCa and there are not enough information about maximal tolerated doses and drug exposure. Therefore, we introduce a toxicity function depending on *ipilimumab* concentration.

Up to our knowledge, in literature there are few mathematical models describing drug toxicity function, and the most detailed has been introduced by Hadjiandreou et al. [21]. The authors described the toxicity as a linear function of the drug concentration multiplied by a coefficient, representing the side effect magnitude. This coefficient has been defined by considering the side effects registered into the patient population, weighted by their severity. However, often adverse events can be considered negligible for low drug doses, while they become more frequent and strong in case of high doses, showing a non-linear relation with drug concentration. Therefore, we use a non-linear toxicity to fit toxicity coefficients, computed from literature experimental observations. For a given clinical protocol, the corresponding toxicity coefficient is defined by the percentage of patients showing moderate and severe adverse events, weighted by a function of the average resolution times of the side effects. The occurrence of side effects can produce a stop of the treatment affecting its efficacy. The need of side effects recovery leads to either a delay or a definitive stop of the treatment delivery. Thus the evaluation of the side effects recovery time is essential in evaluating the drug toxicity. Indeed, in patient populations with equivalent adverse events and frequencies, the life quality of those patients having lower average resolution times considerably improves, and therefore we can take the resolution time as an indication of a a lower drug toxicity.

The paper is organized as follows. In Section 2, we describe the experimental data used to estimate the toxicity function, we present the mathematical model and some technical aspects related to the optimization. We show the model results in Section 3, including the estimates of the toxicity function and the proposed optimal administration protocols, obtained by fixing different constraints. In Appendix B, we also analyze the synergy between the *ipilimumab* and *sipuleucel-T*, by means of a modified Bliss combination index, in order to further investigate how the two immunotherapies work in combination. In the Discussion we summarize the main contributions, linking the mathematical results with biological observations.

## 2. Materials and Methods

### 2.1. Experimental Data

In order to evaluate the toxicity of the drug *ipilimumab*, we consider experimental data from literature. We select the only two clinical trials on PCa patients involving a single infusion of *ipilimumab* [22] and a dose-escalating administration [23], in order to compare different administration protocols. The study by Slovin et al. compares different doses of *ipilimumab* administered every 3 weeks, for 4 cycles. The authors collected the percentages of Adverse Events (AEs) for patients treated with *ipilimumab* alone and in combination with external-beam radiotherapy, but we select only the data regarding the *ipilimumab* administered as mono-therapy. The study by Small et al. considers patients treated with a single dose of *ipilimumab*. The authors collected the frequencies of the single side effects and observed AEs of grade 3 in 2 over 14 patients. This study does not report the percentages of AEs of grade 1 and 2, but, comparing all these data, we are able to derive a range of patients presenting moderate AEs. All these information are summarized in Table 1.

In our analysis, the resolution times of the symptoms is an essential information to evaluate the drug toxicity. However, no one of the selected studies refers the average resolution times of the side effects. To extrapolate this information, we consider the studies on melanoma, which is one of the cancer for which *ipilimumuab* has been approved. In these studies, the average resolution times have been registered only in case of immune-related AEs, and they change depending on the sample. In melanoma patients immune-related AEs are the most common side effects of *ipilimumab* treatments [24], and this is confirmed for PCa patients [23]. Therefore, we selected the studies from Weber et al. [25] and Hodi et al. [26], both administering 3 mg/kg of *ipilimumab* every 3 weeks for 4 cycles, and we set the resolution times of moderate and severe AEs as an average between the ones registered in these studies, obtaining $t_{M}=38.15$ days and $t_{S}=45.15$ days, respectively.

### 2.2. Mathematical Model

**Figure 1:**
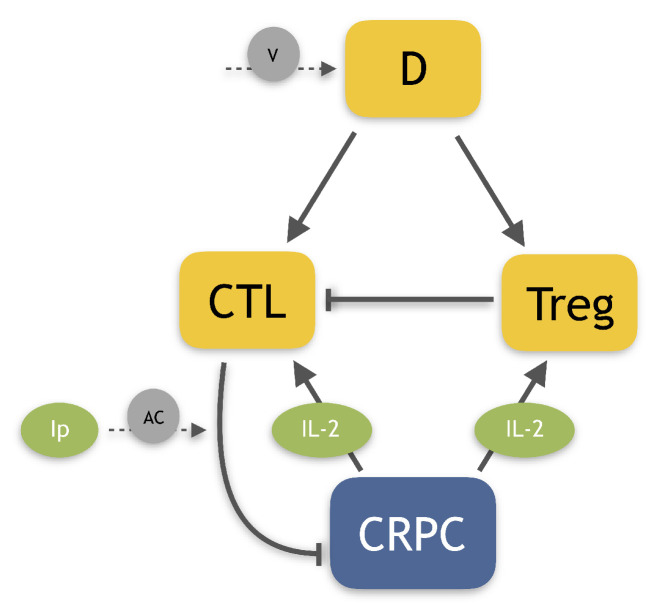
Human PCa model diagram. Cells are represented in orange squared boxes, while molecules in green rounded ones. The Castration-Resistant Prostate Cancer (CRPC) has been highlighted by a blue box. Other players involved: Dendritic cells (D), regulatory T cells (Treg), Cytotoxic T Lymphocytes (CTL), Interleukin-2 (IL-2) and *ipilimumab* ($I_{p}$). The lines represent promotions and inhibitions, while dashed lines depict the treatments: dendritic cell vaccine (V) and anti-CTLA4 drug (AC).

We employ a mathematical model of tumor growth, interacting with the immune system, developed in our previous work [15]. The original model is composed by 8 ordinary differential equations (ODEs), describing the two forms of PCa, (castration sensitive and resistant, previously named androgen dependent and independent) and some key variables of the immune system, such as dendritic cells, Cytotoxic T cells (CTL), Regulatory T cells (Treg), the interleukin-2 (IL-2), as well as the evolution of the Prostate Specific Antigen (PSA) as linear combination of the PCa cells. The model also includes three treatments: the androgen deprivation therapy, the dendritic cell vaccine *sipuleucel-T* and the anti-CTLA4 *ipilimumab*. Since we aim at evaluating the *ipilimumab* efficacy on castration-resistant prostate cancer, we set the androgen deprivation therapy as the mainstay treatment, such that the androgen dependent PCa form can be ignored (CSPC≈0). Moreover, by imposing the quasi-steady state approximation on the variable IL-2, we can further reduce the model, obtaining the following ODE system:

(1) $$\left\{\begin{array}{c} \dot{T}=r\left(1-\frac{T}{K}\right)T-e_{C}\;\frac{C\;T}{s_{C}+T}-k_{AC}\;I_{p}\;C\;T,\\ \dot{C}=e_{D}\frac{D}{s_{D}+D}-\mu_{C}\;C+e_{IL}\frac{C\;I_{L}\left(T,C\right)}{s_{IL}+I_{L}\left(T,C\right)}-k_{R}\;R\;C,\\ \dot{R}=a_{R}\;D-\mu_{R}\;R+a_{IL}\;I_{L}\left(T,C\right),\\ \dot{D}=\rho_{D}-\mu_{D}\;D,\\ \dot{I_{p}}=-\lambda \;I_{p}, \end{array}\right.$$

where *T*, *C*, *R*, *D* and $I_{p}$ represent, respectively, CRPC cells, CTLs, Tregs, dendritic cells and *ipilimumab* concentration, while $I_{L}\left(T,C\right)=i_{0}+e\frac{C\;T}{s+T}$ describes the dynamics of IL-2, obtained by the quasi-steady state approximation. Table A4 in the Appendix D summarizes the parameter estimates. Figure 1 shows a schematic representation of the model-variable interactions.

The CRPC cells are assumed to grow according to a logistic term, while they can be killed by CTLs. The effect of the drug *ipilimumab* has been described by an additional term which increases the CTL tumor-killing capacity (the last term into the first equation). The CTLs are activated by the dendritic cells, and can also increase in number as effect of the clonal expansions due to IL-2. Treg cells repress the immune reaction by reducing the number of activated T cells. These immune cells are activated either by dendritic cells or IL-2. The dendritic cell vaccine has been described by an infusion of dendritic cells.

The starting point of all the model simulations has been set according to the initial condition of the patients in the Slovin et al. study. The authors considered a sample of patients previously treated with androgen deprivation therapy, that have developed to the castration-resistant state. To reproduce the initial condition of this sample, we simulate the complete system in [14] by imposing androgen deprivation therapy, and we find the time $\hat{t}$ such that $PSA(\hat{t})$ corresponds to the average baseline value recorded in the patient population. Then, we choose as initial conditions for (1) the values of the variables of that system at time $\hat{t}$, namely:

$$T=47\;\mathrm{billions}\;\mathrm{of}\;\mathrm{cells}\;C=2.63\times 10^{-5}\;\mathrm{billions}\;\mathrm{of}\;\mathrm{cells}$$

$$R=2.53\;\mathrm{billions}\;\mathrm{of}\;\mathrm{cells}\;\;D=4.9\times 10^{-2}\;\mathrm{billions}\;\mathrm{of}\;\mathrm{cells}\;I_{p}=0\;\mathrm{mg}$$

### 2.3. Computation of Toxicity Coefficients

In order to quantify the toxicity of a given protocol, on the basis of clinical observations, we introduce the coefficients associated to moderate and severe AEs as:

(2) $$C_{k}=\left(1-\frac{1}{t_{k}}\right)g_{k}\;p_{k},\;k=M,\;S;$$

where $C_{M}$ and $C_{S}$ represent the coefficient of toxicity of moderate and severe AEs, $t_{M}=38.15$ and $t_{S}=45.15$ are the average resolution times of the symptoms expressed in days computed as described in Section 2.1, $g_{M}$ and $g_{S}$ are the severity grades and $p_{M}$ and $p_{S}$ are the percentages of patients presenting the side effects. Table 1 provides the values of $p_{M}$ and $p_{S}$ with respect to the severity grades ($g_{M}=1.5$ and $g_{S}=3.5$), in different administration protocols. In this representation, adverse effects with resolution time of 1 day do not contribute to the toxicity coefficient. Higher resolution times increase the value of the term $\left(1-\frac{1}{t_{k}}\right)$, so that the corresponding $C_{k}$ grows. Finally, we define the toxicity coefficient of a given administration protocol *j* as:

(3) $$C_{Tox}^{j}=\frac{C_{M}+C_{S}}{\hat{Tox}},$$

where $\hat{Tox}$ represents the maximal toxicity value, i.e., the coefficient computed in case of all patients having severe AEs, with very high average resolution time such that $\frac{1}{t_{k}}\approx 0$. Therefore, the toxicity coefficients are values between 0 and 1.

By considering the experimental data described in Section 2.1, and by assuming that the times for resolution of AEs are comparable between prostate cancer and melanoma patients, we can compute the toxicity coefficients corresponding to these administration protocols. The results are reported in Table 2.

### 2.4. Toxicity Function

The aim of this work is to find an optimal protocol for *ipilimumab* administration by balancing the efficacy of a therapy in reducing tumor and the drug toxicity. To this end, we need to define a toxicity function for any feasible protocol of *ipilimumab* administration. We assume that the toxicity is a function depending on the drug concentration all over the time of the treatment, namely:

(4) $$Tox\left(I_{p}\right)=\int_{0}^{t_{f}}\frac{aI_{p}{\left(t\right)}^{n}}{b+I_{p}{\left(t\right)}^{n}}dt,$$

The shape of the function depends on the parameters *a*, *b* and *n* that are determined by fitting the function (4) to the experimental data, as described in the Appendix A. In any case, the toxicity is 0 if no *ipilimumab* is administered, increases as the doses are larger and has a bounded value for any possible protocol, consistently with the definition of toxicity coefficient.

### 2.5. Optimization Problems

We define optimal administration protocols for *ipilimumab* those that minimize a weighted combination of average tumor size and overall toxicity over an appropriate time interval, that we choose as 5 years. We computed the optimal protocols, both for the case of administration of *ipilimumab* in mono-therapy and in combination with *sipuleucel-T*.

The optimal controls have been computed under several constraints, which have been suggested by clinical practice, and have also the effect of reducing the computational burden. Precisely, we assumed that the therapy period cannot be longer than 6 months and that there is a minimal interval between treatments, either 1 week or 3 weeks (the typical interval in clinical trials).

The minimal (0.3 mg/kg) and maximal (10 mg/kg) doses have been fixed according to the *ipilimumab* doses tested by clinical trials [23,27,28]. Moreover, we assumed that the *ipilimumab* concentration has to remain below a threshold $I_{p}^{MAX}$, for all the treatment period. The threshold $I_{p}^{MAX}$ has been established on the basis of the maximal-dose clinical trial, i.e. one infusion of 10 mg/kg every 3 weeks for four cycles. Simulating the system for a patient of 88.5 kg (average patient weight according to Coletti et al. approximation [15]), we found the maximal concentration reached and set it as the threshold $I_{p}^{MAX}=1266.55$ mg.

Mathematically, finding the optimal protocol consists in solving the minimum problem defined by the following equation:

(5) $$\min_{\begin{array}{c} d=[d_{1},\ldots ,d_{26}]\\ d_{i}\in \left[0,d_{max}\right],\;i=1,\ldots ,n_{26} \end{array}}\left[w_{1}\int_{0}^{t_{f}}T\left(t\right)dt+w_{2}Tox\left(Ip\left(d,t\right)\right)\right],$$

where $t_{f}=5$ years, and the weights $w_{1}=1$ and $w_{2}=500$ have been empirically fixed in order to make comparable the two terms of the objective function $O_{1}=\int_{0}^{t_{f}}T\left(t\right)dt$ and $O_{2}=Tox\left(Ip\left(d,t\right)\right)$. The First Optimization Problem (OP1) returns the optimal dose-scheduling in terms of the vector $d=[d_{1},\ldots ,d_{26}]$ where $d_{i}$ represents the dose (expressed in multiples of the minimal dose, ranging between 0 and 33) administered at week *i* (26 are the possible weeks in the 6-month period). Each dose varies from 0 to $d_{max}$, where $d_{max}=10$ mg/kg corresponds to the maximum tested dosage (according to the references in Table 1). This problem consists in a combinatorial optimization, which is well suited for genetic algorithms. Therefore, the solutions have been obtained by the *Genetic Algorithm* of Matlab [29,30], implementing a *MustiStart* approach, in order to explore all the parameter space. For each problem, we find an optimal administration protocol for the *ipilimumab*, administered as mono-therapy or in combination with *sipuleucel-T*, administered by following FDA suggestions, i.e., three infusions of 50 million of cells, one every 2 weeks. The minimum problems are repeated by imposing different minimum time intervals between two infusions, i.e., $\Delta_{t}=1$ week and $\Delta_{t}=3$ weeks, as discussed above.

We also consider a Second Optimization Problem (OP2), in which we add the constraint that $T(T_{f})=0$, i.e., that the tumor is eradicated at the end of the treatment. Note that the solutions of (1) mathematically are never exactly equal to 0. However since T(t) in (1) is measured in billions of cells, we set it equal to 0 at $\bar{t}$ if $T\left(\bar{t}\right)<10^{-9}$.

## 3. Results

### 3.1. Toxicity Function Estimates

**Figure 2:**
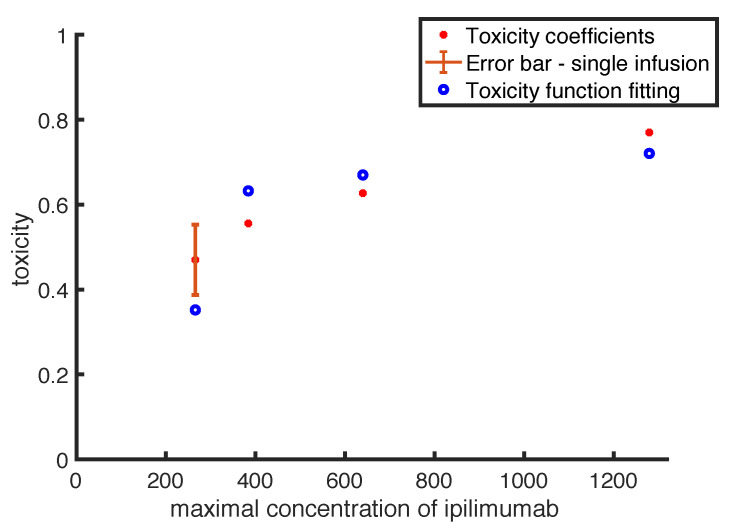
Comparison between toxicity coefficients (red filled points) and the outputs of the toxicity function (blue empty points) evaluated reproducing the same clinical protocols described in Table 3. The point related to the single infusion is represented with the relative error-bar.

The toxicity function defined in Equation (4) needs to be calibrated. Figure 2 shows the fitting between function outputs and toxicity coefficients, which has been obtained by following the procedure presented in Section 2.4. The chart shows with red filled points the values of the *ipilimumab* toxicity coefficients listed in Table 2, with the relative error bar in case of the single-infusion. The blue empty points represent the values of toxicity predicted by the toxicity function. The data points represent different administration procedures: the point with error bar is the toxicity coefficient in case of a single infusion of *ipilimumab*, while the others refer to intermittent protocols with different doses. Therefore, in order to easily compare the data fitting, we put on x-axis the maximal concentration of the drug *ipilimumab*.

Overall, our function provides a good qualitative fit to the data. In particular, the toxicity function reproduces quite well the coefficient values in case of intermittent protocols, in which the error is between 0.04 and 0.08. The worst fit has been obtained in case of single-infusion (the point including error-bar), with an error of 0.1.

The estimates of the parameters in Equation (4) are reported in Table 3.

### 3.2. Optimal Administration Protocols

**Figure 3:**
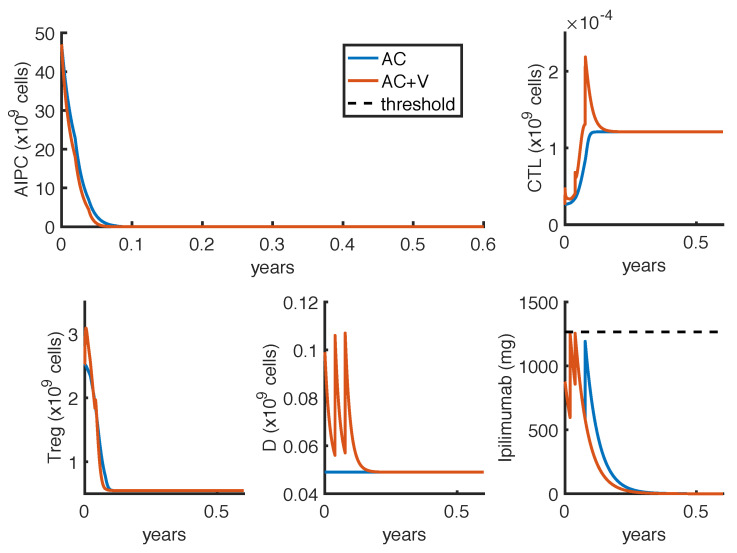
Administration protocol as result of the optimization problem OP1, with $\Delta_{t}=1$ week. Blue lines represent the model dynamics in case of *ipilimumab* mono-therapy (AC), while orange lines represent the one in case of *ipilimumab* and *sipuleucel-T* combination therapy (AC + V). The *ipilimumab* threshold has been highlighted with a black dotted line.

**Figure 4:**
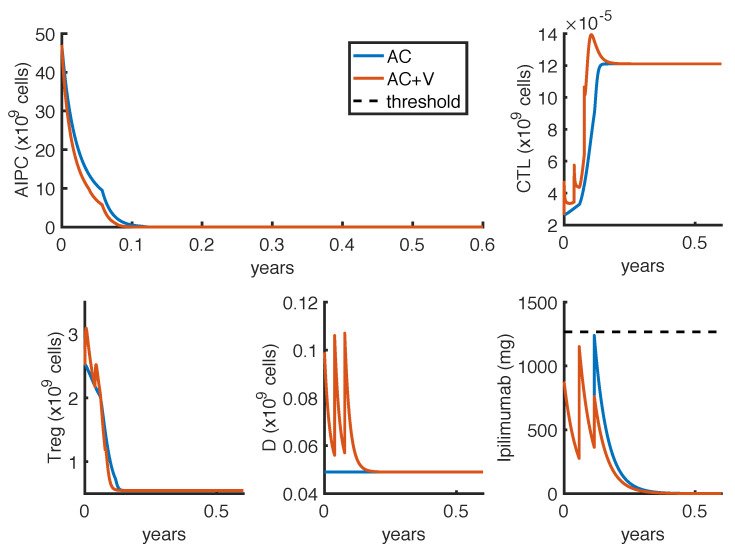
Administration protocol as result of the optimization problem OP1, with $\Delta_{t}=3$ weeks. Blue lines represent the model dynamics in case of *ipilimumab* mono-therapy (AC), while orange lines represent the one in case of *ipilimumab* and *sipuleucel-T* combination therapy (AC + V). The *ipilimumab* threshold has been highlighted with a black dotted line.

As described in Section 2.5, we defined two optimization problems. The first one (OP1) finds the less-toxic anti-CTLA4 schedule able to reduce tumor growth. Figure 3 and Figure 4 show the results obtained by considering, respectively, $\Delta_{t}=1$ week or $\Delta_{t}=3$ weeks, as the minimum time interval between two infusions. The charts compare the outcomes in case of mono-therapy with *ipilimumab* (AC) and combination therapy with *ipilimumab* and *sipuleucel-T* (AC + V). These numerical results are summarized in Table 4.

In all cases, the optimal administration protocols are predicted to control prostate cancer over the therapy period. The optimal schedules strictly depend on the constraint of the minimum time interval $\Delta_{t}$. Indeed, the model suggests to administer high doses of *ipilimumab* as soon as possible, provided that the drug concentration remains under the threshold. This implies that, in case of mono-therapy, when $\Delta_{t}=1$ week, the optimal administration times are at weeks $t_{1}=0,\;t_{2}=1,\;t_{3}=3$ and $t_{4}=4$ (Figure 3), while, when $\Delta_{t}=3$ weeks, the optimal administration times are at weeks $t_{1}=0,\;t_{2}=3$ and $\;t_{3}=6$ (Figure 4). Changing the minimum time between drug administrations, the model suggests two approaches of drug administration: one with more frequent low doses infusions (Figure 3), the other with fewer infusions of high doses (Figure 4). Compared to the mono-therapy, the combination therapy AC + V is predicted to reduce more rapidly the tumor mass, involving a lower dosage of *ipilimumab*, and thus causing a lower toxicity.

**Figure 5:**
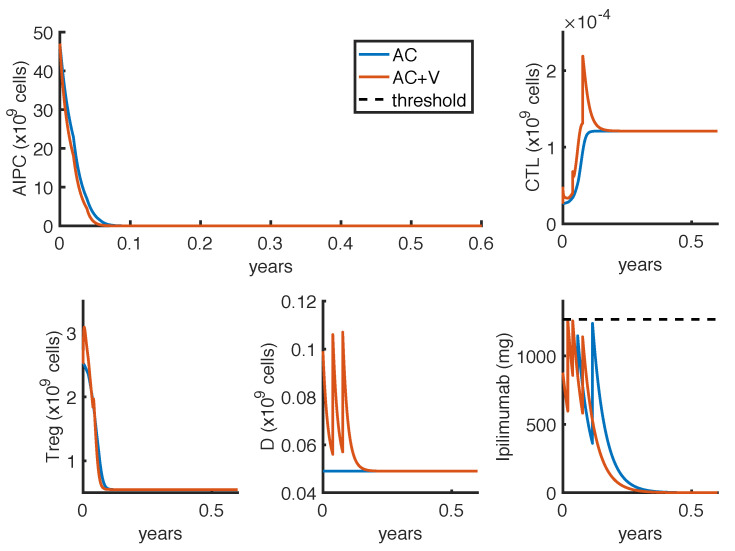
Administration protocol with the aim of tumor eradication as result of the optimization problem OP2, with $\Delta_{t}=1$ week. Blue lines represent the model dynamics in case of *ipilimumab* mono-therapy (AC), while orange lines represent the one in case of *ipilimumab* and *sipuleucel-T* combination therapy (AC + V). The *ipilimumab* threshold has been highlighted with a black dotted line.

**Figure 6:**
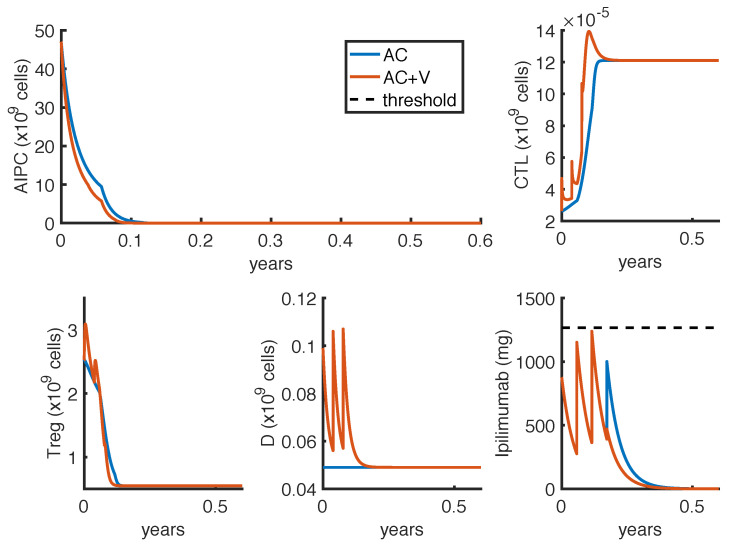
Administration protocol with the aim of tumor eradication as result of the optimization problem OP2, with $\Delta_{t}=3$ weeks. Blue lines represent the model dynamics in case of *ipilimumab* mono-therapy (AC), while orange lines represent the one in case of *ipilimumab* and *sipuleucel-T* combination therapy (AC + V). The *ipilimumab* threshold has been highlighted with a black dotted line.

The other optimization problem OP2 finds the optimal protocol able to eradicate the tumor. Figure 5 and Figure 6 show the model suggested administration protocols in case of two different constraints about the minimum time interval between two infusions of either $\Delta_{t}=1$ week or $\Delta_{t}=3$ weeks, respectively. The charts compare the mono-therapy with anti-CTLA4 (AC) with the effect of the combination therapy with anti-CTLA4 and vaccine (AC + V). The results are presented in Table 5.

As observed for the other optimization problem, the optimal protocol depends on the fixed minimum time interval between two infusions ($\Delta_{t}$), and the model suggests to administer anti-CTLA4 as soon as possible. In this case, some doses are slightly increased reltively to what shown in Table 4, and an additional infusion is necessary in order to eradicate the tumor. The results with the combination therapy confirm the possibility of tumor eradication by using a reduced amount of *ipilimumab*.

### 3.3. Sensitivity of Results to Parameter Estimates

**Figure 7:**
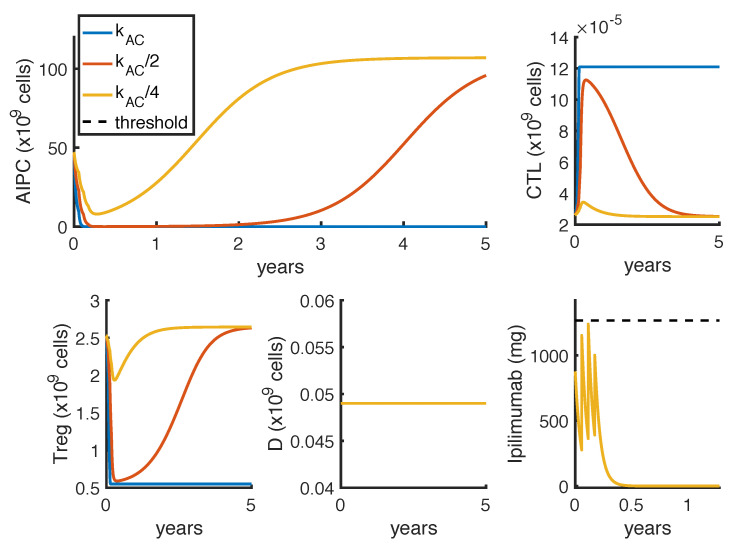
Graphical sensitivy analysis on the parameter $k_{AC}$, representing the maximal killing rate of tumor by CTL due to the drug *ipilimumab*. Blue, orange and yellow lines represent, respectively, the model dynamics by setting the value of $k_{AC}$ as 5.44 (the original estimate), 2.72 ($\frac{k_{AC}}{2}$) and 1.81 ($\frac{k_{AC}}{4}$). The *ipilimumab* threshold has been highlighted with a black dotted line.

The computed results are influenced by the model parameter estimates. In particular, the value of $k_{AC}$, representing the maximal killing rate of tumor by CTL due to the drug *ipilimumab*, strongly affects the efficacy of the proposed administration protocols. The value of this parameter has been estimated in our previous work [15], by fitting experimental data referring to those prostate cancer patients having the greatest benefits from the *ipilimumab* therapy. Therefore, the value estimated for $k_{AC}$ is higher than what adequate for the average of the prostate cancer patient population, and all the model results represent the optimal case of well-responding patients. In order to assess the sensitivity of the results to the value of $k_{AC}$, we analyze the model dynamics for lower values of this parameter. Fixing the optimal administration protocol for the mono-therapy showed in Figure 6, we perform a sensitivity analysis on $k_{AC}$. Figure 7 shows how the model outcomes change as the parameter value is reduced.

These results highlight that by halving $k_{AC}$, even if the administration protocol is able to initially reduce the tumor, the prostate cancer starts increasing again 2 years after the therapy start (orange line). If the parameter value is further reduced, the same therapy is not able to control the tumor growth (yellow line).

**Figure 8:**
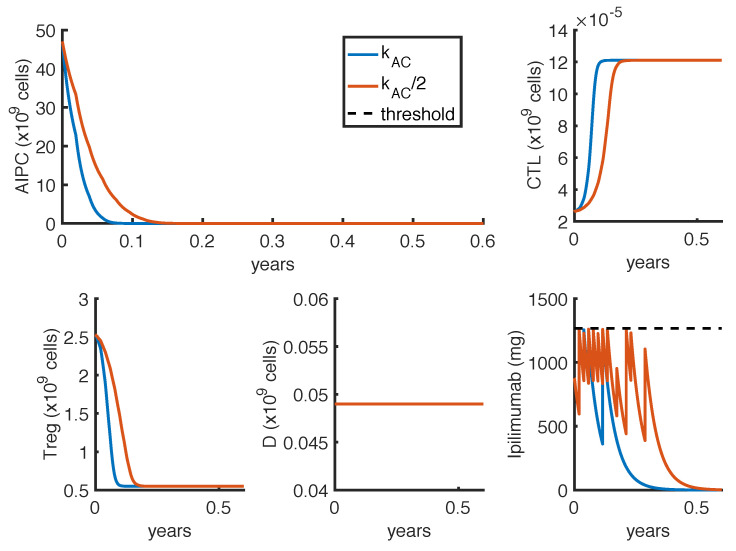
Comparison between administration protocols obtained with different values of the parameter $k_{AC}$. The dynamics refer to the *ipilimumab* mono-therapy as result of the optimization problem OP2, with $\Delta_{t}=1$ week. Blue lines represent the model outcomes with the estimate value of $k_{AC}$ (the same presented in Figure 5), while orange lines represent the one with $\frac{k_{AC}}{2}$. The *ipilimumab* threshold has been highlighted with a black dotted line.

Given the strong influence of the parameter $k_{AC}$ on the results, we investigate if tumor eradication could be possible if its value is half the reference value (Table A4). Therefore, we repeat the optimization procedures presented in Section 2.5 with the reduced value of $k_{AC}$. Figure 8 shows the model suggested optimal protocols in case of mono-therapy with $\Delta_{t}=1$ week. The figure compares the optimal administration protocols by considering the estimate value $k_{AC}$ with the one obtained with $k_{AC}/2$.

**Figure A1:**
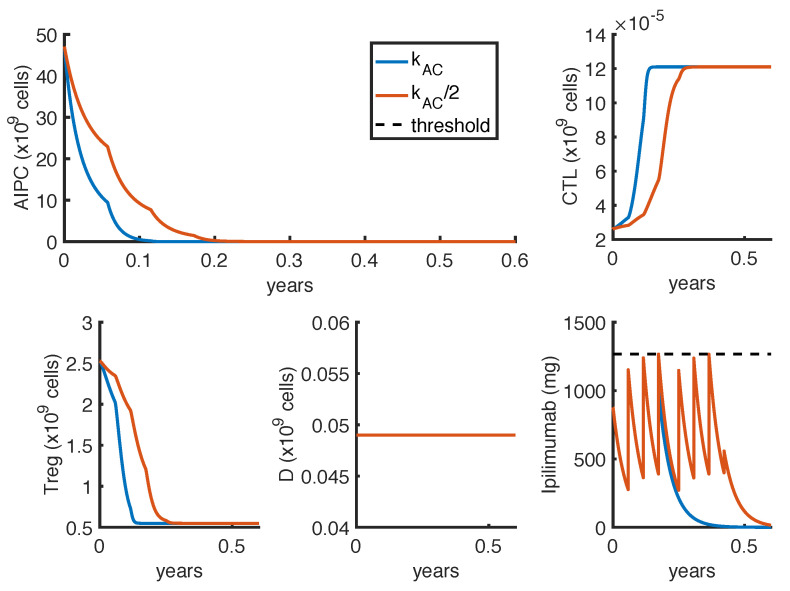
Comparison between administration protocols obtained with different values of the parameter $k_{AC}$. The dynamics refer to the *ipilimumab* mono-therapy as result of the optimization problem OP2, with $\Delta_{t}=3$ weeks. Blue lines represent the model outcomes with the estimate value of $k_{AC}$ (the same presented in Figure 6, blue line), while orange lines represent the one with $\frac{k_{AC}}{2}$. The *ipilimumab* threshold has been highlighted with a black dotted line.

**Figure A2:**
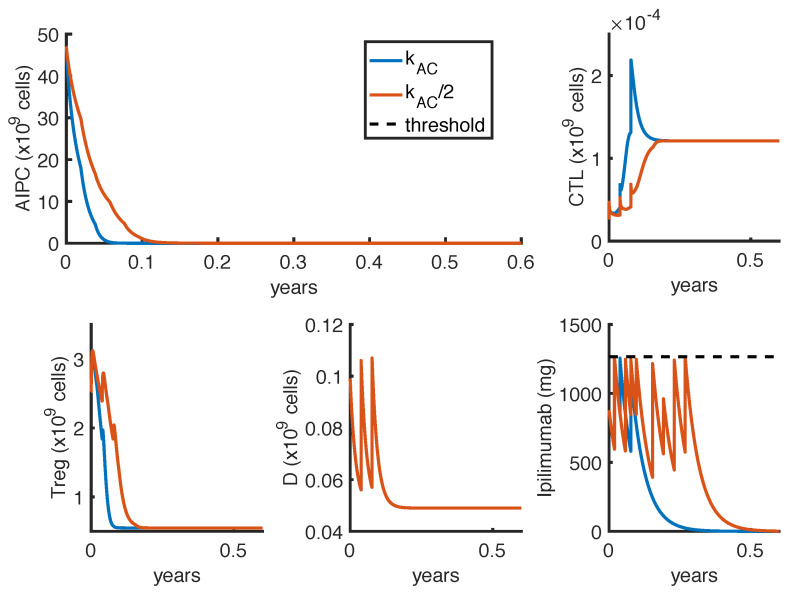
Comparison between administration protocols obtained with different values of the parameter $k_{AC}$. The dynamics refer to the *ipilimumab* and *sipuleucel-T* combination therapy as result of the optimization problem OP2, with $\Delta_{t}=1$ week. Blue lines represent the model outcomes with the estimate value of $k_{AC}$ (the same presented in Figure 5, orange line), while orange lines represent the one with $\frac{k_{AC}}{2}$. The *ipilimumab* threshold has been highlighted with a black dotted line.

**Figure A3:**
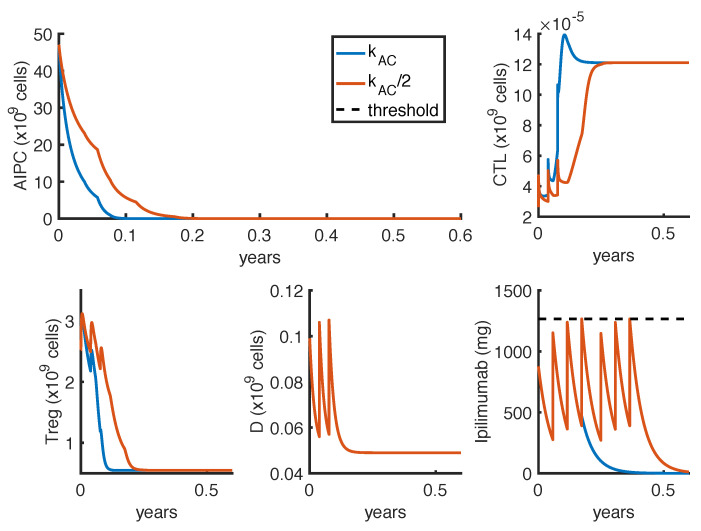
Comparison between administration protocols obtained with different values of the parameter $k_{AC}$. The dynamics refer to the *ipilimumab* and *sipuleucel-T* combination therapy as result of the optimization problem OP2, with $\Delta_{t}=3$ weeks. Blue lines represent the model outcomes with the estimate value of $k_{AC}$ (the same presented in Figure 6, orange line), while orange lines represent the one with $\frac{k_{AC}}{2}$. The *ipilimumab* threshold has been highlighted with a black dotted line.

We repeated the optimization procedure also by setting $\Delta_{t}=3$ weeks (see Appendix C, Figure A1) and for the combination therapy with *ipilimumab* and *sipuleucel-T*, with both $\Delta_{t}=1$ week (Figure A2) and $\Delta_{t}=3$ weeks (Figure A3).

The model predicts that the tumor eradication is possible even if the value of the parameter $k_{AC}$ is half the reference. However, the amount of administered anti-CTLA4 drug is substantially increased, as well as the length of the therapy. By comparing Figure 8 and Figure A1, we observe that by fixing $\Delta_{t}=1$ week the number of *ipilimumab* infusions needed to eradicate the tumor is higher than the one suggested with $\Delta_{t}=3$ weeks. In the first case, the model suggests to administer as much drug as possible, one dose per week for the first two months. After this period, the administrations could be a bit relaxed. On the other hand, by imposing the constraint of $\Delta_{t}=3$ weeks, the predicted optimal administration protocol is constituted by two standard protocols (one infusion per week for 4 cycles) repeated with a one month break.

Similarly to the previous cases, when the dendritic cell vaccine is combined with the anti-CTLA4, the model suggests to administer a lower amount of *ipilimumab* compared to the mono-therapy optimal protocols (Figure A2 and Figure A3). In particular, when $\Delta_{t}=1$ week, the administered doses of anti-CTLA4 needed to eradicate the tumor passes from 12 for the mono-therapy to 9 for the combination therapy. The numerical results for the optimal administration protocols in case of reduced $k_{AC}$ are listed in Table 6. By fixing the parameter $k_{AC}$ at 1/4 of the estimated value reported in Table A4, the model predicts that tumor eradication is no longer feasible with 6-month therapies.

To understand how the parameter uncertainty affects model results, we investigate how the proposed administration protocols vary by perturbing the parameter of the toxicity function. To this end, we implement the optimal control problem by increasing or reducing the parameter *a* of the Equation (4). Our results show that the model predictions are not influenced by the parameter uncertainty, since the optimal control outcomes remain identical after moderate perturbations of a parameter. The predicted optimal administration protocol changes only if the value of *a* is multiplied at least by a factor 5 or divided at least by a factor 2. Even in those cases, the model suggestions remain in line with the ones obtained with the original parameters estimates, proposing always high doses over a short time period, instead of a low-dose and prolonged therapy. Note that changing the weights in the optimization objectives (5) is equivalent to multiplying or dividing the toxicity function. Thus these results can be viewed also as a sensitivity analysis on the weights.

## 4. Discussion

In this work, we employ a mathematical model of human prostate cancer in order to determine optimal *ipilimumab* administration protocols able to reduce/eradicate tumor by balancing the treatment ability in reducing tumor and the drug toxicity. *Ipilimumab* is an anti-CTLA4 approved for the treatment of several tumors, and tested in metastatic castration-resistant prostate cancer [10]. We consider patients previously treated with androgen deprivation therapy, who develop the castration-resistant prostate cancer form, and we investigate the efficacy of immunotherapies on those tumor cells. We examine the effect of *ipilimumab* administered as mono-therapy and in combination with the dendritic cell vaccine *sipuleucel-T*, administered following the FDA recommendations. Our results highlight that the administration of *ipilimumab* is potentially able to control or eradicate the tumor. In particular, the optimal administration protocols seem to be feasible, and the corresponding toxicity profile is comparable with those observed in the clinical trials (Table 2, Table 4 and Table 5), suggesting that the proposed therapy could be well-tolerated. Moreover, the result obtained by fixing $\Delta_{t}=3$ weeks for the optimal administration of *ipilimumab* as mono-therapy can be compared to the high-dose tested protocol [23,28].

The combination with the vaccine is predicted to improve the efficacy of the mono-therapy, since the corresponding optimal protocol provides a faster tumor reduction, while administering a lower amount of anti-CTLA4, with a consequent reduction in *ipilimumab*-related toxicity. This effect is probably due to the increase in CTLs proliferation induced by the dendritic cell vaccine [31], which, coupled with a drug aiming at increasing the CTL tumor-killing activity, causes a stronger tumor suppression. However, clinical studies highlight that only few patients show an evident positive effect as a consequence of *ipilimumab* therapies. In the study by Small et al., only 2 out of 14 patients treated with a single dose of *ipilimumab* had a significant PSA reduction (>50%), while Slovin et al. [23] observed a complete and a partial response in 2 out of 16 patients, treated with intermittent protocols of high-doses. These different outcomes can be explained by patient heterogeneity, since the success of a therapy depends on patient-specific parameters. One of these is the parameter $k_{AC}$, which measures how much the *ipilimumab* can improve the tumor-killing activity of CTLs. As shown in Figure 7, a perturbation of this parameter can qualitatively change the model outcomes, and the optimal protocol becomes unable to even control the tumor growth.

To further investigate if the tumor eradication could be possible even for those patients who respond less well to the standard therapies, we find the optimal protocol for lower values of $k_{AC}$. By analyzing the optimal administration protocols we obtained under different model constraints on $\Delta_{t}$, i.e., the minimum time interval between two infusions, we see that the model exhibits two different administration approaches: either one with frequent infusions at lower dosage, or another one consisting in two repeated standard protocols (one infusion every 3 weeks for 4 cycles) with maximal doses. Both the values of $\int_{t_{1}}^{t_{f}}I_{p}\left(t\right)dt$ (tumor density) and $Tox(t_{f},I_{p}\left(t\right))$ (toxicity) corresponding to the choice $\Delta_{t}=3$ weeks are higher than the ones obtained by imposing $\Delta_{t}=1$ week (Table 6). Similar results hold for the reference value of $k_{AC}$ (Table 4 and Table 5), although the difference is lower. However, we cannot conclude that the optimal protocol with $\Delta_{t}=3$ weeks is less suitable than the one with $\Delta_{t}=1$ week, since our results need to be validated from the clinical point of view and our analysis does not consider side effects and practical constraints. On the one hand, we do not have any information about close-range *ipilimumab* infusions, and therefore the numerical results obtained with $\Delta_{t}=1$ week could not be reliable, since a patient could be affected by an increased toxicity depending on frequent infusions, which are not taken into account. Furthermore, we neglect the cost of the drug administration, which is, however, a crucial information to determine the feasibility of a therapy. For instance, comparing the mono-therapy protocols (rows 1 and 3 in Table 6), the one associated to $\Delta_{t}=1$ week consists in administering 12 infusions of *ipilimumab*, while the other one with $\Delta_{t}=3$ weeks consists in 8 infusions. As every infusion of *ipilimumab* costs around $30,000 [32], the predicted optimal administration protocol with $\Delta_{t}=1$ week, in reality, could not be feasible.

Our results point out the synergy between *ipilimumab* and *sipuleucel-T* (see Appendix B). By looking at the optimal protocols, the vaccine allows one to reduce the anti-CTLA4 dosage while reaching the goal of tumor eradication. The vaccine administered alone shows poor performances, while, coupled with the *ipilimumab*, its effectiveness considerably improves. The limited effect of the vaccine has been observed in other clinical studies [31], which highlight a few months increase in life expectancy, but not a PSA reduction. This could suggest to administer the dendritic cell vaccine in combination with other immunotherapies. Moreover, the results showed in Table A1 and Table A3 predict a stronger synergy for lower values of $k_{AC}$. This seems indicate that a combination therapy could provide best performances for those patients who do not have a good response to *ipilimumab* mono-therapy. The potential synergy between *ipilimumab* and *sipuleucel-T* has also been observed in clinical studies, such as [33], which report an increase of the median survival in patients with metastatic-progressive prostate cancer. A phase 1 clinical trial is currently investigating the efficacy of this combination immunotherapy in patients with advanced prostate cancer [34]. Thought a sensitivity analysis on a toxicity function parameter, we also investigate the reliability of the model prediction, by evaluating if the model outcomes can be highly influenced by the uncertainty in computing drug toxicity. Our analysis indicates that the computed optimal administration protocols seems not be dramatically affected by parameter perturbation, by highlighting a general robustness of the analysis herein reported.

In conclusion, the presented work is a theoretical analysis of the effect of *ipilimumab* under optimal protocols of administration. Additional efforts are needed to make this approach suitable to clinical applications. The definition we used for the toxicity function could be more accurate if more detailed experimental data were available. For example, we assumed that the resolution times of AEs of *ipilimumab* in PCa can be compared with the ones registered in melanoma. However, the median age of melanoma patients is usually lower than the one of PCa patients, and this could affect the resolution times. Moreover, the resolution time corresponding to the single infusion of *ipilimumab* was supposed to be the same as for the intermittent administration protocol, due to the lack of this information in the study by Small et al. [22]. When patient’s treatment time series and associated clinical data will be publicly available, it would be also interesting to generate an in silico population of patients to compare the outcomes of the optimal control problem with clinical trial results, in terms of individual patient responses. It is important to note that our results depend on the initial state used for the model simulations. In the perspective of proposing a personalized protocol, the initial state should be set in accordance with the patient condition. The model can also be calibrated to patient-specific parameters, such as the tumor proliferation rate. In this perspective, the model outcomes can be used not only to find the optimal protocol for a personalized therapy, but also to evaluate if the patient responds well to the treatment. Indeed, by comparing the model predicted tumor progression with the patient prostate cancer evolution, the model could indicate, in almost real time, whether the treatment is working as expected, and therefore could be helpful in supporting clinical decisions.

## 5. Conclusions

In this paper, we employ a previously developed mathematical model of castration-resistant prostate cancer, in order to determine optimal protocols for the administration of the drug *ipilimumab*. This work provides a theoretical study about the efficacy of *ipilimumab*, working alone or in combination with the dendritic vaccine *sipuleucel-T*. Our model predicts that *ipilmumab* is potentially able to eradicate prostate cancer. Moreover, our results highlight a synergy between *ipilimumab* and *sipuleucel-T*, which allows for a reduction of the amount of *ipilimumab* administered, with a stronger effect on tumor reduction. Other efforts are necessary to employ our methods in a clinical environment, but we think that this theoretical work can help in understanding the potential effectiveness of the drug *ipilimumab*. Moreover, we hope that our results could encourage the scientific community to further investigate the role of immunotherapy agents in the treatment of advanced prostate cancer.

## Figures and Tables

**Table 1:** Percentages of AEs registered in prostate cancer patients subject to *ipilimumab* therapy [22,23]. The two columns Grade 1–2 AEs and Grade 3–4 AEs represent the magnitude of the side effects, which is grade 1 and 2, and grade 3 and 4, respectively. The data are expressed in terms of number of patients and corresponding percentages ($p_{M}$ and $p_{S}$ in the Equation (2)). In Small et al., severe AEs were only of grade 3, and their number is clearly reported, while we infer a possible range of patients presenting AEs of grade 1 and 2.

	Grade 1–2 AEs	Grade 3–4 AEs
	*n* (%)	*n* (%)
Slovin et al.		
3 mg/kg	6 (75)	2 (25)
5 mg/kg	2 (33)	3 (50)
10 mg/kg	6 (37)	10 (63)
Small et al. min-max	7–12 (50–85.7)	2 (14.3)

**Table 2:** Toxicity coefficients computed by considering the data reported in Table 1. $C_{M}$ and $C_{S}$ represent the coefficients of toxicity of moderate and severe adverse events employed to evaluate the toxicity coefficient ($C_{Tox}$) for a given protocol by the Equation (3). The coefficient of the single *ipilimumab* dose is represented by its average value ± standard deviation, since the corresponding reported data allow us to only estimate a possible range of values.

*Ipilimumab* Administration Protocol	$C_{M}$	$C_{S}$	$C_{\mathrm{Tox}}$
single dose of 3 mg/kg	0.99 (±0.26)	0.42	0.47 (±0.09)
3 mg/kg every 3 weeks (4 cycles)	1.09	0.86	0.56
5 mg/kg every 3 weeks (4 cycles)	0.48	1.71	0.63
10 mg/kg every 3 weeks (4 cycles)	0.54	2.14	0.77

**Table 3:** Parameter estimates of the toxicity function in Equation (4).

Parameter	Estimate	Unit of Measure
*a*	$4.02\;\times \;10^{-3}$	-
*b*	4.66	mg
*n*	2	-

**Table 4:** Numerical results for the optimization problem OP1. Anti-CTLA4 has been administered as mono-therapy (AC) and in combination with dendritic cell vaccine (AC + V). Both the immunotherapies have been tested by taking into account the general constraints defined in Section 2.5 and an additional constraint concerning the minimum time interval between two infusions ($\Delta_{t}$) fixed as 1 or 3 weeks. Column three and four list the infusion times and doses predicted by the model as the optimal schedules. The values $O_{1}$ and $O_{2}$ represent the two components of the optimization function, namely $O_{1}=\int_{t_{1}}^{t_{f}}Tum\left(t\right)dt$ and $O_{2}=Tox\left(t_{f},I_{p}\left(t\right)\right)$.

Therapy	$\Delta_{t}$	Infusion Times (Weeks)	Doses (mg/kg)	$O_{1}$	$O_{2}$
AC	1 week	$t_{1}=0,\;t_{2}=1,$ $t_{3}=2,\;t_{4}=4$	$d_{1}=10,\;d_{2}=7.5,$ $d_{3}=4.5,\;d_{4}=7$	354.47	0.57
AC + V	1 week	$t_{1}=0,\;t_{2}=1,$ $t_{3}=2$	$d_{1}=10,\;d_{2}=7.5,$ $d_{3}=4.5$	292.67	0.52
AC	3 weeks	$t_{1}=0,\;t_{2}=3,$ $t_{3}=6$	$d_{1}=10,\;d_{2}=10,$ $d_{3}=10$	483.01	0.63
AC + V	3 weeks	$t_{1}=0,\;t_{2}=3$ $t_{3}=6$	$d_{1}=10,\;d_{2}=10$ $d_{3}=4.5$	371.13	0.60

**Table 5:** Numerical results for the optimization problem OP2. Anti-CTLA4 has been administered as mono-therapy (AC) and in combination with dendritic cell vaccine (AC + V). Both the immunotherapies have been tested by taking into account the general constraints defined in Section 2.5 and an additional constraint concerning the minimum time interval between two infusions ($\Delta_{t}$) fixed as 1 or 3 weeks. Column three and four list the infusion times and doses predicted by the model as the optimal schedules. The values $O_{1}$ and $O_{2}$ represent the first two components of the optimization function, namely $O_{1}=\int_{t_{1}}^{t_{f}}Tum\left(t\right)dt$ and $O_{2}=Tox\left(t_{f},I_{p}\left(t\right)\right)$.

Therapy	$\Delta_{t}$	Infusion Times (Weeks)	Doses (mg/kg)	$O_{1}$	$O_{2}$
AC	1 week	$t_{1}=0,\;t_{2}=1,$ $t_{3}=2,\;t_{4}=3,$ $t_{5}=6$	$d_{1}=10,\;d_{2}=7.5,$ $d_{3}=4.5,\;d_{4}=3,$ $d_{5}=10$	350.83	0.63
AC + V	1 week	$t_{1}=0,\;t_{2}=1,$ $t_{3}=2,\;t_{4}=4$	$d_{1}=10,\;d_{2}=7.5,$ $d_{3}=4.5,\;d_{4}=6$	279.23	0.57
AC	3 weeks	$t_{1}=0,\;t_{2}=3,$ $t_{3}=6,\;t_{4}=9$	$d_{1}=10,\;d_{2}=10,$ $d_{3}=10,\;d_{4}=7$	482.06	0.70
AC + V	3 weeks	$t_{1}=0,\;t_{2}=3$ $t_{3}=6,\;t_{4}=9$	$d_{1}=10,\;d_{2}=10$ $d_{3}=10,\;d_{4}=1$	367.79	0.65

**Table 6:** Numerical results for the optimization problem OP2, with a halved value of $k_{AC}$. Anti-CTLA4 has been administered as mono-therapy (AC) and in combination with dendritic cell vaccine (AC + V). Both the immunotherapies have been tested by taking into account the general constraints defined in Section 2.5 and an additional constraint concerning the minimum time interval between two infusions ($\Delta_{t}$) fixed as 1 or 3 weeks. Column three and four list the infusion times and doses predicted by the model as the optimal schedules. The values $O_{1}$ and $O_{2}$ represent the first two components of the optimization function, namely $O_{1}=\int_{t_{1}}^{t_{f}}Tum\left(t\right)dt$ and $O_{2}=Tox\left(t_{f},I_{p}\left(t\right)\right)$.

Therapy	$\Delta_{t}$	Infusion Times (Weeks)	Doses (mg/kg)	$O_{1}$	$O_{2}$
AC	1 week	$\begin{array}{l} t_{1}=0,\;t_{2}=1,\\ t_{3}=2,\;t_{4}=3,\\ t_{5}=4,\;t_{6}=5,\\ t_{7}=6,\;t_{8}=7,\\ t_{9}=9,\;t_{10}=11,\\ t_{11}=12,\;t_{12}=15 \end{array}$	$\begin{array}{l} d_{1}=10,\;d_{2}=7.5,\\ d_{3}=4,\;d_{4}=5,\\ d_{5}=4.5,\;d_{6}=4,\\ d_{7}=5,\;d_{8}=4.5,\\ d_{9}=4,\;d_{10}=9,\\ d_{11}=4,\;d_{12}=8 \end{array}$	667.95	0.88
AC + V	1 week	$t_{1}=0,\;t_{2}=1,$ $t_{3}=3,\;t_{4}=4,$ $t_{5}=5,\;t_{6}=8,$ $t_{7}=10,\;t_{8}=12$ $t_{9}=14$	$d_{1}=10,\;d_{2}=7.5,$ $d_{3}=7.5,\;d_{4}=4.5,$ $d_{5}=4.5,\;d_{6}=9,$ $d_{7}=4.5,\;d_{8}=9$ $d_{9}=8$	574.09	0.86
AC	3 weeks	$t_{1}=0,\;t_{2}=3,$ $t_{3}=6,\;t_{4}=9,$ $t_{5}=13,\;t_{6}=16,$ $t_{7}=19,\;\;t_{8}=22$	$d_{1}=10,\;d_{2}=10,$ $d_{3}=10,\;d_{4}=10,$ $d_{5}=10,\;d_{6}=10,$ $d_{7}=10,\;d_{8}=2$	1002.52	1.03
AC + V	3 weeks	$t_{1}=0,\;t_{2}=3,$ $t_{3}=6,\;t_{4}=9,$ $t_{5}=13,\;t_{6}=16,$ $t_{7}=19\;$	$d_{1}=10,\;d_{2}=10,$ $d_{3}=10,\;d_{4}=10,$ $d_{5}=10,\;d_{6}=10,$ $d_{7}=10$	809.54	1.00

## Data Availability

The data are all included in the manuscript.

## Abbreviations

The following abbreviations are used in this manuscript:

PCa	Prostate cancer
CSPC	Castration Sensitive Prostate Cancer
CRPC	Castration Resistant Prostate Cancer
AC	Anti-CTLA4 therapy
V	Dendritic cell vaccine therapy
FDA	Food and Drug Administration
CTL	Cytotoxic T Lymphocytes
Treg	Regulatory T cells
PSA	Prostate Specific Antigen
OP1	First Optimization Problem
OP2	Second Optimization Problem

## Appendix A. Toxicity Function Parameter Estimation

In order to determine the values of the parameters *a*, *b* and *n* of the Function (4), we implement a minimum problem aiming at reducing the squared distance between the toxicity coefficients and the function outputs:

(A1) $$\min_{\left[a,b,n\right]}\;{\left(||Tox\left(\left[a,b,n\right],I_{p}\right)-C_{Tox}{\left|\right|}_{2}\right)}^{2},$$

where $C_{Tox}=\left[C_{Tox}^{1},C_{Tox}^{2},C_{Tox}^{3},C_{Tox}^{4}\right]$ is a vector containing the *ipilimumab* toxicity coefficients in Table 2, and $Tox(\left[a,b,n\right],I_{p})$ is the function defined in the Equation (4) evaluated reproducing the corresponding clinical protocols. We observe that the toxicity function could be written as $Tox\left(\left[a,b,n\right],I_{p}\right)=aF\left(\left[b,n\right],I_{p}\right)$, with $F\left(\left[b,n\right],I_{p}\right)=\int_{t_{i}}^{t_{f}}\frac{I_{p}{\left(t\right)}^{n}}{b+I_{p}{\left(t\right)}^{n}}dt$. This allow us to find the optimal estimate for *a* as function of the others, reducing the number of the parameters to estimate for the minimum problem in Equation (A1). Indeed, if $G\left(\left[a,b,n\right],I_{p}\right)={\left(||Tox\left(p,I_{p}\right)-C_{Tox}{\left|\right|}_{2}\right)}^{2}$, then

(A2) $$\frac{\partial G(\left[a,b,n\right],I_{p})}{\partial a}=2\sum_{j}\left(aF^{j}\left(\left[b,n\right],I_{p}\right)-C_{Tox}^{j}\right)F^{j}\left(\left[b,n\right],I_{p}\right).$$

By imposing $\frac{\partial G(\left[a,b,n\right],I_{p})}{\partial a}\geq 0$, we obtain the optimal value:

(A3) $$a^{*}=\frac{\sum_{j}C_{Tox}^{j}\;F^{j}\left(\left[b,n\right],I_{p}\right)}{\sum_{j}{\left(F^{j}\left(\left[b,n\right],I_{p}\right)\right)}^{2}}.$$

Therefore, the new minimum problem becomes:

(A4) $$\min_{\left[b,n\right]}\;{\left(\left|\right|a^{*}F\left(\left[b,n\right],I_{p}\right)-C_{Tox}{\left|\right|}_{2}\right)}^{2}.$$

The parameter *b* and *n* have been estimated, respectively, between real and natural numbers in $\left[1,\;10^{30}\right]$ and [1,10], and the corresponding values are listed in Table 3.

## Appendix B. Synergy

Synergy To investigate the synergy between the immunotherapies included in the model, we consider the Bliss combination index [35,36], which has been successfully employed to analyze the drug synergies in other studies [13,14]. This index compares the efficacy of the combination therapy, with the one obtained by considering the two drugs as not interacting, computed by probability laws. To do this, we define the efficacy of a treatment *j* as the resulting tumor reduction due to a therapy in comparison with the untreated case ($T^{sx}$):

(A5) $$E\left(j\right)=\frac{T^{sx}\left(t_{f}\right)-T^{j}\left(t_{f}\right)}{T^{sx}\left(t_{f}\right)},$$

where *j* is the therapy, while $t_{f}=5$ years, is the time at which we evaluate the tumor reduction. The value of E(j) is in [0,1], where 0 means that the treatment *j* does not lead to a tumor reduction, while 1 indicates that $T^{j}\left(t_{f}\right)=0$, i.e., the cancer is eradicated. Therefore, we define a slightly modified Bliss combination index as:

(A6) $$\sigma \left(AC,V\right)=\frac{E(AC,V)}{E(AC,0)+E(0,V)-E(AC,0)\cdot E(0,V)}-1,$$

where E(AC,V) is the efficacy of the combination therapy including anti-CTLA4 and vaccine, while E(AC,0) and E(0,V) are the efficacies of the two mono-therapies. If σ(AC,V)>0, this means that the two drugs are synergistic, otherwise, if σ(AC,V)<0, the two drugs are antagonistic.

Since the synergy defined in Equation (A6) depends on the treatment efficacies, we evaluate the combination indexes by varying the *ipilimumab* dose (Table A1). The synergy index is always positive, therefore the two drugs are predicted to be synergistic, but it first increases with *ipilimumab* dose, by reaching its maximal value for infusions of 3 mg/kg, and then starts decreasing.

This behavior could be easily explained by looking at the treatment efficacies, defined by Equation (A5). Indeed, since the model predicts a low effectiveness of the vaccine administered as mono-therapy, with $E\left(0,V\right)=1.98\cdot 10^{-9}$, the denominator of the Equation (A6) E(AC,0)+E(0,V)−E(AC,0)E(0,V)≈E(AC,0). Therefore, the synergy index becomes a comparison between the the efficacy of the combination-therapy and the *ipilimumab* mono-therapy. Table A2 summarizes the model predicted efficacies of the *ipilimumab* mono-therapy according to the different doses. We observe that increasing the dose the estimated treatment efficacy tends to 1, which means that PCa after 5 years is close to zero, and therefore the addition of the dendritic cell vaccine does not lead to a significant improvement.

**Table A1:** Index of synergy between *sipuleucel-T* (sip-T in the table) and *ipilimumab* for different *ipilimumab* doses, computed by the Formula (A6). The two drugs are administered by following the standard procedures. Anti-CTLA4 is administered every 3 weeks, for 4 cycles, while the vaccine is performed by infusing three doses of 50 million of cell every 2 weeks.

*ipilimumab* dose (mg/kg)	0.3	1.5	2.1	3.0	3.6	4.5	5.1	6	7.5
Synergy with sip-T (unitless)	0.11	0.38	0.89	1.78	0.37	8.9 $\times \;10^{-3}$	7 $\;\times \;10^{-4}$	1.54 $\;\times \;10^{-5}$	2.2 $\;\times \;10^{-16}$

**Table A2:** Model predicted efficacy of the *ipilimumab* mono-therapy for different doses, computed by the Formula (A5). The drug is administered every 3 weks, for 4 cycles.

*ipilimumab* dose (mg/kg)	0.3	1.5	2.1	3.0	3.6	4.5	5.1	6	7.5
Predicted efficacy (unitless)	7.86$\;\times \;10^{-5}$	1.32$\;\times \;10^{-3}$	5.64 $\;\times \;10^{-3}$	0.13	0.67	0.99	0.99	1.00	1.00

These results are influenced by the estimate of $k_{AC}$, therefore we further analyze the synergy by considering a reduced value of this parameter. Table A3 shows the synergy indexes between *sipuleucel-T* and *ipilimumab* and the model predicted efficacies for the anti-CTLA4 mono-therapy for different *ipilimumab* doses.

**Table A3:** Index of synergy between *sipuleucel-T* (sip-T in the table) and *ipilimumab* and anti-CTLA4 mono-therapy efficacy for different *ipilimumab* doses and an halved value of $k_{AC}$. The coefficients of synergy and efficacy are computed by the Equations (A5) and (A6), respectively. The two drugs are administered by following the standard procedures. Anti-CTLA4 is administered every 3 weks, for 4 cycles, while the vaccine is performed by infusing three doses of 50 million of cell every 2 weeks.

*ipilimumab* dose (mg/kg)	0.3	1.5	2.1	3.0	3.6	4.5	5.1	6	7.5
Synergy with sip-T (unitless)	0.1	0.2	0.2	0.4	0.6	1.1	1.6	1.8	0.2
Predicted efficacy (unitless)	3.5$\;\times \;10^{-5}$	2.8$\;\times \;10^{-4}$	5.3$\;\times \;10^{-4}$	1.3$\;\times \;10^{-3}$	2.6$\;\times \;10^{-3}$	8.8 $\;\times \;10^{-3}$	2.4$\;\times \;10^{-2}$	0.1	0.8

By comparing Table A1 and Table A3, we observe that the model predicts a stronger synergy with a halved value of $k_{AC}$ than with the estimated parameter.

## Appendix D. Parameters Estimates

**Table A4:** Table of parameter estimates for the model.

Parameter	Description	Estimate	Reference
*r*	Proliferation rate of CRPC cells	$0.006\;{\mathrm{day}}^{-1}$	[37]
*K*	Tumor carrying capacity	107 billion of cells	[15]
$e_{C}$	Maximal killing rate of tumor by CTL in untreated case	0.75 day${}^{-1}$	[37]
$s_{C}$	CTL saturation level for tumor cells inhibition	10 billion of cells	[37]
$k_{AC}$	Maximal killing rate of tumor by CTL due to the drug *ipilimumab*	$5.44\;\frac{1}{\mathrm{day}\cdot \mathrm{mg}\cdot \mathrm{billion}\;\mathrm{of}\;\mathrm{cells}}$	[15]
$e_{D}$	Maximal activation rate of CTL by dendritic cells	$0.02\;\frac{\mathrm{billion}\;\mathrm{of}\;\mathrm{cells}}{\mathrm{day}}$	[37]
$s_{D}$	Dendritic cells saturation level for T cell clonal expansion	0.4 billion of cells	[37]
$\mu_{C}$	Death rate of CTL	0.03 day${}^{-1}$	[37]
$e_{IL}$	Maximal activation rate of CTL by IL-2	0.1245 day${}^{-1}$	[37]
$s_{IL}$	IL-2 saturation level for T cell clonal expansion	$1000\;\frac{\mathrm{ng}}{\mathrm{mL}}$	[37]
$k_{R}$	Inactivation rate of CTL by Tregs	$32.81\;\frac{1}{\mathrm{day}\cdot \mathrm{billion}\;\mathrm{of}\;\mathrm{cells}}$	[15]
$a_{R}$	Activation rate of Treg by mature dendritic cells	0.072 day${}^{-1}$	[38]
$\mu_{R}$	Death rate of Treg	0.72 day${}^{-1}$	[38]
$a_{IL}$	Activation rate of Treg by IL-2	$131.26\;\frac{\mathrm{billion}\;\mathrm{of}\;\mathrm{cells}}{\mathrm{day}}\frac{\mathrm{mL}}{\mathrm{ng}}$	[15]
$\rho_{D}$	Source of dendritic cells	$0.00686\;\frac{\mathrm{billion}\;\mathrm{of}\;\mathrm{cells}}{\mathrm{day}}$	[15]
$\mu_{D}$	Death rate of dendritic cells	0.14 day${}^{-1}$	[37]
λ	*Ipilimumab* death rate	0.055 day${}^{-1}$	[15]
$i_{0}$	Baseline level of IL-2	$0.00299\;\frac{\mathrm{ng}}{\mathrm{mL}}$	[15]
*e*	Maximal activation rate of IL-2 by CTL	$5000\;\frac{\mathrm{ng}}{\mathrm{mL}\cdot \mathrm{day}\cdot \mathrm{billion}\;\mathrm{of}\;\mathrm{cells}}$	[37]
*s*	Tumor cells saturation level for CTL stimulation	10billionofcells	[37]
